# Rapid effects of progesterone on ciliary beat frequency in the mouse fallopian tube

**DOI:** 10.1186/1477-7827-8-48

**Published:** 2010-05-15

**Authors:** Anna Bylander, Magdalena Nutu, Rikard Wellander, Mattias Goksör, Håkan Billig, DG Joakim Larsson

**Affiliations:** 1 Institute of Neuroscience and Physiology, the Sahlgrenska Academy, University of Gothenburg, SE-405 30 Göteborg, Sweden; 2 Department of Physics, University of Gothenburg, SE-412 96 Göteborg, Sweden

## Abstract

**Background:**

The physiological regulation of ciliary beat frequency (CBF) within the fallopian tube is important for controlling the transport of gametes and the fertilized ovum. Progesterone influences gamete transport in the fallopian tube of several mammalian species. In fallopian tubes isolated from cows, treatment with 20 micromolar progesterone caused a rapid reduction of the tubal CBF. The aims of this study were to establish methodology for studying fallopian tube CBF in the mouse, as it is an important model species, and to investigate if progesterone rapidly affects the CBF of mice at nM concentrations.

**Methods:**

A method to assess tubal CBF of mice was developed. Fallopian tubes were dissected and the tissue was cut in small pieces. Tissue samples with moving cilia were located under an inverted bright field microscope and held still against the bottom of a petri dish by a motorized needle system. Images were acquired over 90 minutes at 35 degrees C with a high-speed camera and used for assessing changes in the CBF in response to the addition of hormone.

**Results:**

The baseline CBF of the mouse fallopian tube was 23.3 +/- 3.8 Hz. The CBF was stable over at least 90 minutes allowing establishment of a baseline frequency, addition of hormone and subsequent recordings. Progesterone at concentrations of 20 micromolar and 100 nM significantly reduced the CBF by 10% and 15% respectively after 30 minutes compared with controls.

**Conclusions:**

The present study demonstrates that the mouse, despite its small size, is a useful model for studying the fallopian tube CBF ex vivo. The rapid reduction in CBF by 100 nM progesterone suggests that gamete transport in the fallopian tube could be mediated by progesterone via a non-genomic receptor mechanism.

## Background

The epithelium of the mammalian fallopian tube consists mainly of two cell types, ciliary and secretory cells. The transport of gametes is aided by ciliary movements and muscular contractions where the cilia seem to play a dominant role [1,2]. Regulation of ciliary motility is therefore important to facilitate the meeting of gametes and the subsequent transport of the fertilized ovum to its implantation site. Several factors have been reported to affect tubal ciliary motility including progesterone (P_4_) [3-6], estradiol [3], interleukin 6 [7], prostaglandins [8,9], angiotensin II [10] and Ca^2+ ^[11].

Progesterone has been proposed to regulate ciliary beat frequency (CBF) in the fallopian tube of several mammalian species [3-6]. For example, treatment with P_4 _(10 μM) reduced the CBF of humans by 40-50% 24 hours post treatment ex vivo [3]. The effect of P_4 _was reversed by RU486, a nuclear progesterone receptor antagonist, suggesting an action via the classical nuclear receptor [3]. To our best knowledge, possible rapid effects of P_4 _on fallopian tube CBF have yet not been studied in humans. However, Wessel et al. showed that 20 μM of P_4 _reduced the CBF by 11% within 15 minutes in dissected fallopian tubes from cows. Furthermore, this effect was not reversed by RU486, which supports the involvement of another receptor than the nuclear progesterone receptor [12].

Possible mediators of these effects are the membrane progesterone receptors (mPRα, β and γ) initially described by Zhu et al. [13,14]. Upon P_4 _stimulation, mPRs have been proposed to regulate oocyte maturation [15,16], gonadotropin secretion [17], control of myometrial functions [18] and sperm motility [19] via interactions with G-proteins. Our previous reports demonstrated the presence of mPRβ and mPRγ in the ciliated epithelial cells of the mouse and human fallopian tube, proposing an involvement of these receptors in the control of tubal ciliary activity in these species [20,21]. In the mouse fallopian tube the expression of both receptors was regulated after P_4 _administration [21].

Although there are many studies on tubal ciliary activity in larger mammals such as cows [12], humans [3] and rabbits [22], we have not been able to find any studies on fallopian tube CBF in mice. This species would be valuable to study, as their general physiology is well studied and the mouse provide superior opportunities for studying the involvement of different gene products through the availability of genetically modified strains. The ultrastructure of the fallopian tube is similar between mice and humans, including the distribution of mPRs [20,21]. Despite the considerable difference in length, the duration of egg transport in the fallopian tube is approximately equal between these species [23]. Mice are easy to manipulate and readily available, therefore it would be worthwhile to investigate their utility as models for studying the mechanisms behind P_4_-regulated CBF in the mammalian fallopian tube. It is important to develop models to unravel mechanisms regulating the transport of gametes, as dysfunctions of the fallopian tube might lead to, for example, ectopic pregnancies or other forms of infertility [24].

The first aim of this study was therefore to establish a method to analyze the CBF ex vivo in mice. A second aim was to investigate if P_4 _causes a rapid reduction in CBF similar to the situation in cow [12]. In these earlier studies 20 μM of P_4 _was used. This is a rather high concentration, given that the affinities for progesterone of both nuclear [22] and membrane progesterone receptors [11] are within the nM range. Therefore, we also wanted to investigate possible effects at nM concentrations in the mouse.

## Methods

### Tissue collection and sample preparation

Fallopian tubes were obtained from immature (3.5-5 weeks-old) female C57BL/6 mice from Charles River, Kisslegg, Germany. The animals were kept under a 12:12-h light-dark schedule at 21 ± 2°C with ad libitum access to chow and water. Animals were allowed to acclimate to the animal facilities for ≥ 5 days before initiation of experiment. The mice were killed by cervical dislocation and both fallopian tubes were dissected out and washed in PBS (Phosphate Buffer Saline, pH 7.0). After removal of the broad ligament, fat and blood vessel the fallopian tubes were transferred to petri-dishes containing 37°C pre-warmed G-MOPS™ Plus-medium (Vitrolife, Gothenburg, Sweden). This medium is designed to support the handling and manipulation of oocytes and embryos in an air atmosphere.

Small fallopian tube segments reaching from the ampullary to the infundibulum region were then cut and retransferred to fresh medium. The tube-like segments were longitudinally opened and chopped in smaller pieces. After an additional washing step the samples were kept in medium at 35°C until measurements. The experiments were approved by the local animal ethics committee in, Gothenburg, Sweden (246/2007 and 392/2008) to Joakim Larsson.

The CBF recordings were carried out on tissue samples on the day after sampling at a temperature of about 35°C. The samples were placed in petri dishes (35 mm) with glass bottom microwells (14 mm) (MatTek Corporation, Ashland, USA) containing 2 ml of pre-warmed G-MOPS™. To be able to add hormone and distribute it evenly in the dish while measuring the CBF, the samples must be prevented from moving. After trying out different methods, the best alternative found was pressing a piece of a tissue sample against the bottom of the petri dish using a sterilized needle (TransferTip^®^, iD 15 μm, oD 20 μm, Eppendorf AG, Hamburg, Germany) connected to a motorized microinjection system (TransferMan, Eppendorf). Thus, after locating a piece of tissue with beating cilia the sample was secured to the bottom of the dish by the Eppendorf system. On each piece of tissue, the CBF of at least two different cells that were not adjacent to each other was measured in parallel. Only cells at the perimeter of the tissue were used for analysis.

### Detection system

The CBF was measured using an inverted bright field microscope (Nikon TE-300, Nikon Instruments, Inc, New York) equipped with a 100× oil immersion objective (Numerical Aperture = 1.3). The microscope objective was heated by an adjustable heater loop attached to the objective and regulated by a controller unit (Bioptechs, Butler, Pennsylvania). Images of the ciliary movement were acquired with a 12 bit high speed camera (Prosilica EC1020, Prosilica Inc, Burnaby, Canada) where a region of interest of approximately 50 × 50 pixels could be recorded with a speed of 100 frames per second. The spatial positions of cilia attached to a single cell were determined from the recorded images by a center-of-mass algorithm in LabView (National Instruments, Austin, TX). The center of mass for an image composed of several loose structures such as cilia is a point in space at which the cilia's whole mass can be considered to be concentrated to. This facilitates the calculation of the ciliary movement. Finally, the distribution of beat frequencies present was calculated through a fast Fourier transform (FFT) in which the recorded ciliary movements are decomposed into components of different frequencies.

### Characterization of assay and progesterone exposure

To characterize the assay, individual variability as well as the influence of incubation time on the CBF was investigated. Progesterone (>99% purity, Sigma-Aldrich, St Louis, MO), was first dissolved in ethanol and then through further dilutions steps into G-MOPS™. To add P_4 _to the cells, half of the medium in the dish was replaced with an equal volume of medium containing ethanol and P_4_, resulting in a final exposure concentration of 0.1% (v/v) ethanol and 20 μM or 100 nM P_4_. Control cells were exposed to ethanol (0.1% (v/v)) only. A pilot study was performed measuring the CBF during 20 minutes before and 20 minutes after replacing half of the medium. Similarly, the CBF was also studied in cells exposed to medium with 0.1% (v/v) ethanol for 1.5 h. No clear effect was observed neither by the replacement of medium nor by incubating the cells with ethanol (data not shown).

A baseline frequency for each cell was first assessed based on measurements every 5 minutes for 30 minutes, after which the drug was added together with medium. Then the CBF was recorded every 5 minutes for the following 60 minutes. In the cow, P_4 _affects the CBF within 15 minutes [7]. Our pilot experiments suggested a similar response time in mice, reaching stable values within 30 minutes. To allow a response to develop, the average CBF of the last 30 minutes of the recording period was therefore calculated and compared with the baseline frequency for each cell to assess the effect of added P_4_.

### Statistics

The CBF data for the 20 μM concentration was analyzed from 11 pieces of tissue exposed to progesterone and 8 control pieces exposed to ethanol only derived from in total 11 mice. The Δ frequency before and after treatment for each cell was calculated as the difference between the mean CBF during the last 30 minutes of the measurement and the mean baseline CBF during the first 30 minutes of the measurement. From each piece of tissue, the average Δ frequency of two cell replicates was used. As there was a predefined hypothesis that P_4 _lowers the CBF, a one sided Student's t-test was applied.

For the 100 nM concentration the CBF data were analyzed from 16 pieces of tissue from 8 mice. In this experiment, we used a paired approach as we were able to include both control and progesterone exposed tissues from all animals, thereby improving the statistical power. As the CBF varies between cells, the CBF for each cell was first normalized against its baseline CBF. For each piece of tissue an average CBF at each time point was calculated based on analyses of two cells per tissue. For each mouse the difference in CBF between the tissue exposed to progesterone and the control tissue was calculated. To test the effect of added P_4_, the mean differences for the first 30 minutes and the last 30 minutes were calculated for all eight individuals. As above there was a predefined hypothesis that P_4 _lowers the CBF, thus a one sided paired student's t-test was applied.

## Results

### Characterization of assay

**Figure 1:**
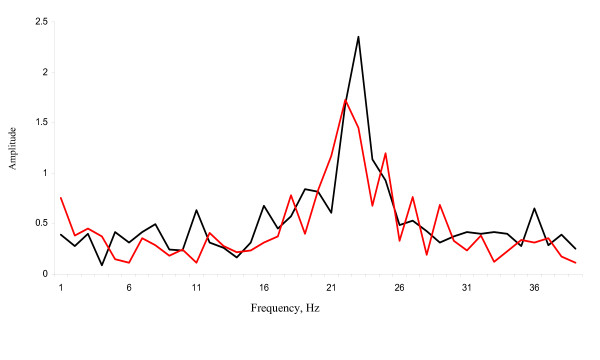
**Example of Fourier-transformed data recording ex vivo of ciliary beat frequency from two ciliated cells from a mouse fallopian tube**.

**Figure 2:**
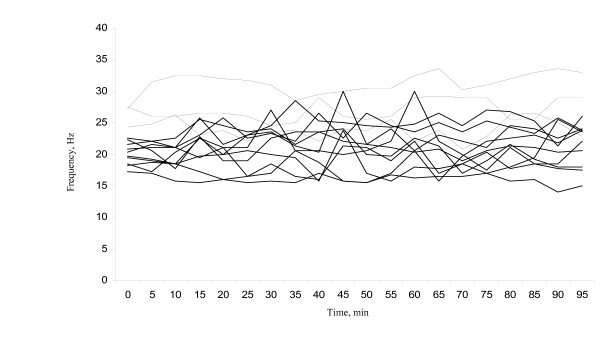
**Ciliary beat frequencies of the control cells measured in the fallopian tube ex vivo of thirteen individual mice**. The frequency was measured with five minute time intervals for 95 min at a temperature of 35°C.

The imaging analyses gave a clear recording which, after Fourier-transformation, could be translated to a CBF (Figure 1). A mean baseline CBF (based on recording over the first 30 minutes) was calculated from 19 mice. The CBF prior to any treatment was 23.3 ± 3.8 Hz (average ± standard deviation) with a range from 16.8 to 31.9 Hz. Figure 2 shows the mean frequency of the control cells from thirteen individuals measured every five minutes for 95 minutes and illustrates the variation that exist between different individuals and over time. Although the CBF differed between mice and between time points for individual mice, there was no clear trend of either a decrease or an increase in CBF during the 95 minute period of the analysis.

### Response to progesterone

**Figure 3:**
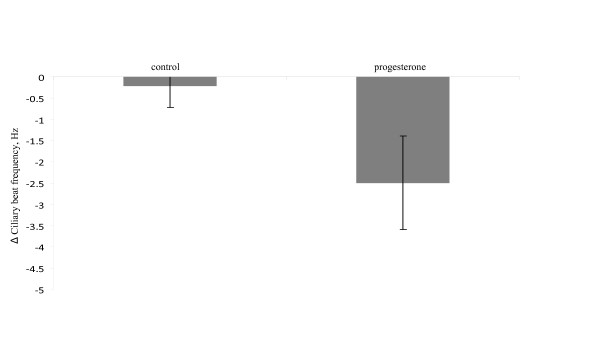
**Mean decrease in beat frequency of ciliated cells from mouse fallopian tubes ex vivo, treated with ethanol only (0.1%; left bar) or with ethanol + progesterone (20 μM; right bar)**. Error bars indicate the standard error of the mean.

**Figure 4:**
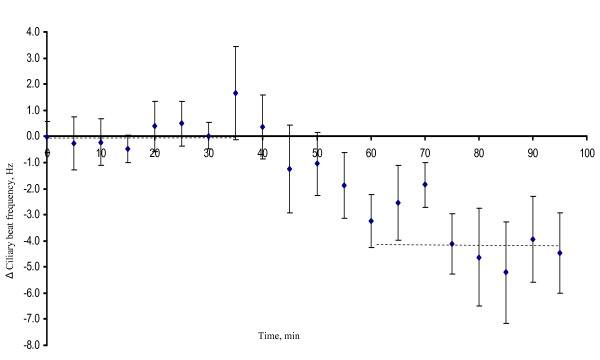
**The average difference in CBF between cells exposed to 100 nM P_4 _and control cells based on sixteen fallopian tube samples from eight mice (paired comparisons)**. The CBF for each cell was first normalized against its baseline CBF. Progesterone was added after 30 minutes. For each individual mouse the difference between cells exposed to P_4 _and control cells was calculated for every time point. The mean differences over the first 30 minutes (baseline period) and the last 30 minutes are shown as crosshatched lines.

Exposure to 20 μM progesterone significantly reduced the mean Δ frequency with 2.28 Hz corresponding to about 10% as compared with cells exposed to ethanol only (p = 0.034). The CBF of control cells exposed to ethanol only was reduced by, on average, 0.2 Hz comparing the end of the recording period with the baseline frequency (Figure 3). This reduction in frequency over time was not significantly different from zero (p = 0.66; one sample t-test). Similarly, 100 nM P_4 _significantly reduced the mean Δ frequency with 3.74 Hz corresponding to about 15% reduction compared to the frequency of the control cells (p = 0.009) (Figure 4).

## Discussion

We have used a light reflection-based method for recording CBF of epithelial cells of the mouse fallopian tube. Given the experimental conditions, the CBF was stable over a period of at least 95 minutes, allowing an establishment of a baseline CBF, addition of hormone, and a subsequent recording of the ciliary activity. Thus, we have established a procedure for studying the effects of different factors on the CBF in the mouse fallopian tube.

The mean CBF was approximately 23.3 ± 3.8 Hz in the mouse. In cows, a mean CBF of 23.1 Hz with a standard deviation of 4.7 Hz was reported [12], whereas in humans the baseline CBF ranges between 5 and 20 Hz [3,25]. In some ex vivo studies, the CBF increased after several hours/days in culture [3,26]. The assessment of the baseline frequency is an important aspect in the evaluation of ciliary activity. A deviation from the normal CBF might in some cases relate to pathological conditions. The experimental conditions, including temperature, also have potential to affect the CBF. A lower assay temperature used in human studies to overcome effects on muscular contractility may partly explain the lower CBF reported for humans compared with both cows [12] and mice (present study).

Wessel et al. used a P_4 _concentration of 20 μM [12]. The same concentration was used in the present mouse study. We found a significantly lowered CBF 30 minutes after addition of 20 μM P_4 _with about 2.28 Hz corresponding to 10% compared with the control cells. This is very similar to the results found in the cow, where P_4 _reduced the CBF by 11% within 15 minutes after adding P_4_. Twenty μM is a high concentration in relation to circulating serum levels, which normally range between 25 - 50 nM in mice [27] and 1 - 10 nM in humans [28]. Although the concentration of P_4 _has not yet been measured in the immediate vicinity of the traveling cumulus complex, it can be expected to be higher than the circulating levels, as the granulosa cells actively produce and secrete P_4 _within the fallopian tube in both mice and humans [29,30]. Indeed, human spermatozoa are attracted to the radially distributed cumulus cells, i.e. granulosa cells surrounding the ovum, by chemotaxis, as these cells produce a P_4 _concentration gradient [31,32]. Thus, associating circulating levels of P_4 _with the change in CBF in the fallopian tube may therefore be of limited value [4,22,25,33-36]. Still one can discuss if 20 μM is a physiological relevant concentration since P_4 _receptors, both PGR (k_d _= 1-5 nM and mPRs (k_d _= 20-30 nM [13]) have affinities for P_4 _in the nM range. Using a concentration considerably higher than the affinity for the receptors might result in unspecific effects. Therefore, we also chose to study the effects of a concentration of P_4 _just above the k_d _of the receptors that may be involved in the response. The CBF was significantly reduced by 3.74 Hz corresponding to 15% 30 minutes after addition of 100 nM P_4_. This reduction of CBF was very similar to the reduction by 20 μM and also to the result seen in cow. Thus, there seem to be important and strong similarities in the response to P_4 _between the cow and the mouse. Given the relatively long evolutionary distance between cow and mouse within the mammalian taxon, this suggests that rapid effects of P_4 _on CBF in the fallopian tube most likely exist in other mammalian species as well.

The stimulation of sperm swimming is another example of rapid P_4 _regulation of ciliary or flagellary activity [19]. Neither in sperm nor in fallopian tube cilia does pre-treatment with a nuclear P_4 _receptor antagonist prevent the rapid effects of P_4_, suggesting an action of P_4 _via different receptor system than the classical nuclear receptors [12,37]. Furthermore the effects of P_4 _on sperm as well as fallopian tube cilia are too rapid to easily be explained by a genomic action, involving transcription and translation. Membrane progesterone receptors (mPR alfa, beta and gamma) have been proposed to mediate these effects [19,20,38-40]. Furthermore, G-proteins that have been reported to mediate P_4 _action via the mPRs in many species and cell systems [41] have been identified in ciliated cells of the fallopian tube [42,43]. The present study is important in this context as it is the first study to link a rapid reduction of fallopian tube CBF by P_4 _with the presence of mPRs in the fallopian tube in the very same species. However, immunostaining to the nuclear progesterone receptor has also been reported on the ciliated cells of the fallopian tube in mice [44]. Interestingly, the staining was restricted to the actual cilia, and no staining was found in the nucleus, suggesting a non-classical function of the receptor. The presence of multiple progesterone receptor types outside of the nucleus provides alternative mechanisms for explaining the rapid actions of progesterone in these cells. Further mice studies with specific selective receptor agonists and antagonists, as well as using transgenic animals, would be useful in elucidating the signaling mechanism of P_4 _action in the fallopian tube of mammals.

## Conclusions

Our study demonstrates that the mouse is a useful model for studying the fallopian tube CBF ex vivo. The rapid reduction in CBF by nM concentrations of progesterone suggests that the effect could be mediated via a non-genomic receptor mechanism, possibly involving mPRs. The similar result seen in both cow and mouse suggests that rapid effects of P_4 _on CBF in the fallopian tube most likely exist in other mammalian species as well.

## Competing interests

The authors declare that they have no competing interests.

## Authors' contributions

AB carried out the tissue preparations and CBF measurements, analysed the data and participated in the writing of the manuscript. MN developed the method for tissue preparation and participated in the writing of the manuscript. RW did the technical set up for the detection system and the computer program for analysing the CBF readings. MG participated in the design and helped to draft the manuscript. DGJL conceived of the study and participated in its design and coordination, performed the statistical analysis and helped to draft the manuscript. MG, HB and DGJL supervised the study. All authors participated in the design of the study, read and approved the final manuscript.
